# Increasing Accessibility to Neuroscience through Translation: Going beyond the English Language

**DOI:** 10.1523/ENEURO.0392-23.2023

**Published:** 2024-01-10

**Authors:** Alba Peris-Yague, Jenna Hartstein, Arielle Hogan, Carla Suhr, Rafael Romero-Calderón

**Affiliations:** ^1^PhD Program in Neuroscience, Universidad Autonóma de Madrid-Cajal Institute, Madrid 28029, Spain; ^2^Laboratory for Clinical Neuroscience, Center for Biomedical Technology, Universidad Politécnica de Madrid, IdISSC, Madrid 28223, Spain; ^3^University of California, Los Angeles, California 90095; ^4^Interdepartmental PhD Program in Neuroscience, University of California, Los Angeles, California 90095; ^5^Department of Spanish and Portuguese, University of California, Los Angeles, California 90095; ^6^Undergraduate Interdepartmental Program in Neuroscience, University of California, Los Angeles, California 90095; ^7^Brain Research Institute, University of California, Los Angeles, California 90095

**Keywords:** communication, education, neuroscience, Spanish, translation

## Abstract

Declaring the 1990s as *The Decade of the Brain* put the field of neuroscience at the forefront of public attention, with the nervous system becoming a subject of increasing interest in popular media. Although this has generally brought large swaths of the public closer to neuroscience, most current research is published and disseminated in a single language: English. This is unsurprising as English is indeed the lingua franca in scientific circles, but people around the world communicate in many other languages. To make neuroscience accessible to a larger audience, we share an initiative to translate the Knowing Neurons platform into a second language: Spanish. This collaborative project integrates humanities and STEM academic programs to make use of bilingual university students, in association with professional linguists and neuroscientists, to translate scientific content into a relatable format to Spanish speakers regardless of their country of origin. The translation effort was piloted within the framework of undergraduate outreach courses at the University of California, Los Angeles, and is coupled with outreach components targeting the Spanish-speaking community to promote this new resource. This project aims to foster an environment where the neuroscientific interests of the public, college students, instructors, and researchers coalesce in a unified space. We hope that opening new lines of communication with traditionally underrepresented communities might help combat the persistent lack of diversity in neuroscience (and STEM) that is currently seen in academia. We also provide an outline to inspire others to translate these, and similar resources, into other languages.

## Significance Statement

Despite the rise in neuroscience-related news in the past few decades, barriers to the integration of neuroscience in social discourse still exist. Scientific illiteracy stemming from the lack of resources generated for public consumption is compounded when considering they are mostly only available in English. Because of this, neuroscience remains an unfamiliar topic to many individuals. Additionally, the humanities and STEM have traditionally been siloed in distinct academic departments providing little opportunity for interdisciplinary collaboration. Our project provides an outline for how to integrate these disciplines to translate neuroscience into a second language, increasing accessibility to a worldwide audience. For the collaborative nature and impact of this project, we were honored with the 2022 Society for Neuroscience Next Generation Award.

## Introduction

The designation of the 1990s as “The Decade of the Brain” by the United States Congress cast a spotlight on the field of Neuroscience (Jones and Mendell, 1999). This led the Society for Neuroscience (SfN) to adopt a new strategic plan in 2003 emphasizing the importance of outreach and neuroscience education (Cameron and McNerney, 2006). In collaboration with other organizations such as the International Brain Organization (IBRO) and the Dana Foundation, initiatives to promote neuroscience to high school and undergraduate students such as the International Brain Bee, the International Youth Neuroscience Association, and Brain Awareness Week (Cameron and McNerney, 2006; Myslinski, 2022) were established. Shortly after, in 2012, the Gatsby and Kavli foundations partnered with SfN to create brainfacts.org, a platform for the general public containing brain-related resources (SfN, 2012). While these neuroscience outreach efforts have been successful, their impact has been restricted because the resources created were exclusively produced in English.

Indeed, English has become the de facto lingua franca in various cross-disciplinary areas, including scientific research (Kushner, 2003), where 98% of scientific publications are written in English (Gordin, 2015). While the use of a single language in scientific circles facilitates communication (Gordin, 2015), it still excludes non-English speaking specialists in the workforce, such as medical doctors or biomedical researchers (Meneghini and Packer, 2007; Silva et al., 2022). Notably, it also excludes large portions of the world population that do not use English to acquire new knowledge.

With this in mind, Knowing Neurons (https://knowingneurons.com), the well-established, nonprofit neuroscience outreach and communication platform, in collaboration with the Department of Spanish and Portuguese and the Brain Research Institute at UCLA, decided to systematically translate all of its content into a second language, namely, Spanish. Spanish is the second-highest spoken language worldwide when ranked by the number of native speakers (474.7 million) and the fourth when second-language speakers are considered (548.3 million; Eberhard et al., 2023). Moreover, the United States is home to the second largest population of Spanish speakers in the world (Carreira, 2013) with 62.5 million Hispanics representing 19% of the population (United States Census Bureau, 2022), and Spanish is the second most spoken language in the country after English (Martínez García and Martínez García, 2022).

Important initiatives within the United States bridging the English- and Spanish-speaking neuroscientific worlds already exist, but they tend to be limited in scope and reach. For instance, Colón-Rodríguez et al. (2019) designed the *Bridge to Neuroscience Workshop*, an outreach program where students in the United States conduct a one-time workshop session for Spanish-speaking high school and college undergraduate students in Puerto Rico, in order to increase neuroscience awareness. Similarly, The Neuroscience Outreach Network (NeurOn), a grassroots organization, also promotes neuroscience in Spanish but mainly focuses on school-aged children (grades K–12; 5–18 years of age) in underserved communities. Other outreach groups have also started to bring neuroscience to Spanish-speaking K–12 and college students but in a more localized fashion, such as the Columbia University Neuroscience Outreach (CUNO) Spanish Education Program and BioBus, both primarily centered around the New York City area. Likewise, initiatives such as *¡Juntos Unidos!*, started at the University of California, San Diego, have been created to provide information, education, and support to the Spanish-speaking Parkinson's disease community. Notably, certain outreach groups are not only conducting some of their community work in Spanish but are also starting to translate some of their resources into Spanish, such as Simply Neuroscience and NW Noggin, in association with the Latino Network; but these efforts are nascent, and the translation work is inadequate for effective communication.

While programs such as these are becoming more common and increase the accessibility of neuroscience resources to more diverse populations, they tend to be age and geographically restricted and do not keep up with the rate of scientific discovery. One notable exception is the Spanish language podcast “Mi Última Neurona,” established in 2021 by the McGovern Institute for Brain Research at MIT, which disseminates the personal and professional trajectories of neuroscientists throughout Latin America. Our project aims to provide an important additional resource in this space to allow a wider swath of Spanish speakers to join the conversation, by generating a Knowing Neurons Spanish-language sister website that publishes cutting-edge research and didactic activities on an ongoing basis. We also provide an outline for a novel academic partnership between the humanities and life sciences at UCLA that is producing high-quality translations of Knowing Neurons content, which can serve as a template to translate these resources into other languages.

## Materials and Methods

### The team

**Figure 1.**
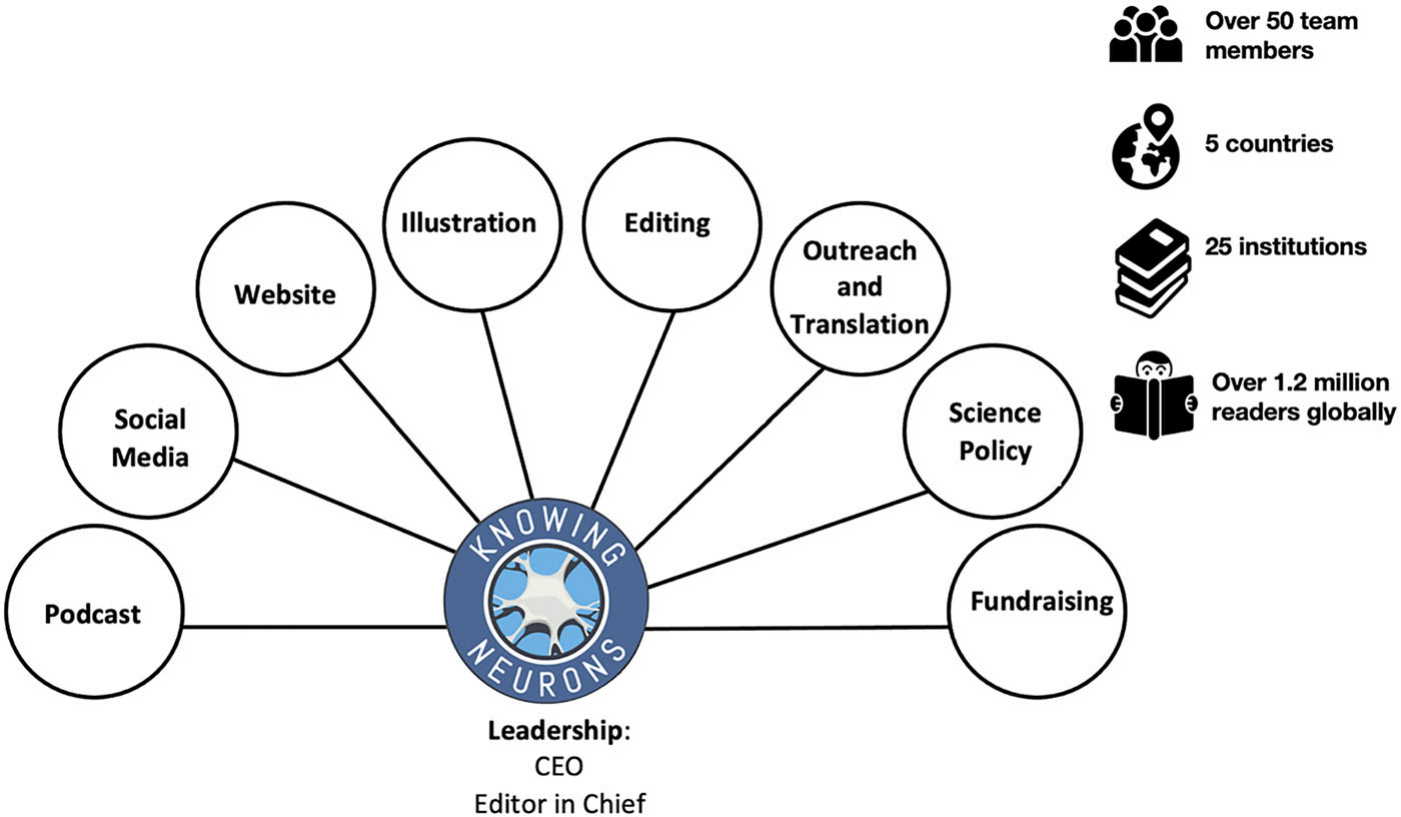
Knowing Neurons structure and organization. Knowing Neurons is led by a chief executive officer and an editor-in-chief who collectively provide the vision for the organization and delegate strategic tasks to teams of graduate student volunteers with the relevant expertise.

Knowing Neurons was created by a group of graduate students at the University of California, Los Angeles (UCLA), and the University of Southern California (USC) in 2012. It is a well-structured organization comprised of a small leadership board, which delegates specific operational and administrative tasks to small teams to manage and maintain the website (Fig. 1). Its mission is to publish widely accessible and accurate articles, podcasts, and infographics on neuroscience concepts and current research, while providing a platform for neuroscience students at various academic levels to train in science communication. Since its inception, it has reached over 1.2 million readers globally and produced over 500 pieces. The team now comprises over 56 neuroscience/biology graduate and undergraduate students from 26 institutions across 5 countries (Table 1). Volunteers come from accredited institutions around the world and although the total number of volunteers fluctuates, there has been a consistent trend towards increasing membership over the last decade. For their contributions to neuroscience education and outreach, the Knowing Neurons team was awarded the Next Generation Award in 2016 by the Society for Neuroscience and again in 2022 for the work described in this paper.

**Table 1.** Knowing Neurons member affiliations

Country	Universities	Members (number)
United States	UCLA	22
	University of Alabama at Birmingham	3
	University of Virginia	3
	Washington University, St. Louis	3
	Harvard University	2
	University of California, San Francisco (UCSF)	2
	University of North Carolina, Chapel Hill	2
	Arizona State University	1
	Broad Institute of MIT and Harvard	1
	Children's National Hospital (Washington, DC)	1
	Louisiana State University	1
	The Ohio State University	1
	Uniformed Services University of the Health Sciences	1
	University of California, Irvine	1
	University of Florida	1
	University of Massachusetts, Amherst	1
	University of Texas, Austin	1
United Kingdom	Imperial College London	2
	Institute of Cancer Research, London	1
	University of Cambridge	1
Spain	Universidad Politécnica de Madrid/Universidad Autónoma de Madrid	1
Italy	University of Parma	1
Germany	Hertie Institute for Clinical Brain Research	1
	University of Potsdam	1
	Würzburg University	1

Number of Knowing Neurons active members by country and university.

The Department of Spanish and Portuguese at UCLA (https://spanport.ucla.edu) is composed of over 25 faculty researching the languages, literatures, and cultures of Latin America and the Iberian Peninsula. It also coordinates the teaching of Spanish as a second language, Spanish for heritage learners, and Spanish for professional purposes for over 3,500 UCLA students per academic year. The Brain Research Institute at UCLA (https://bri.ucla.edu) brings together over 300 neuroscientists from over 30 departments to promote and facilitate research collaborations, education, and outreach. Specifically, these faculty are responsible for teaching the courses for the Neuroscience Major, Minor, and Graduate Program, and their collective expertise includes every aspect of neuroscience research from molecular organization to human behavior.

### Article selection

Our process started by compiling a list of previously published articles from the Knowing Neurons platform and ranking them in order of pedagogical relevance. More precisely, we established priority by referencing the article topics with the subject matter we normally teach in our introductory neuroscience courses and weighing topics considered basic more heavily. We therefore decided to start with Knowing Neurons articles discussing foundational neuroscience concepts categorized under the heading “Fundamentals” and further subclassified them into “Neuro Primers,” “Brain Basics,” “Did You Know?”, and “Neuroscience Technologies.” Once we translated this basic list, we continued with the articles found under the headings “Health,” “Cognition,” and “Beyond,” selectively choosing them in order to cover the widest variety of topics possible (Extended Data 2-3).

### Recruitment of translators

Undergraduate translators were identified through two community outreach courses taught by the Department of Spanish and Portuguese at UCLA. These courses, *Taking it to the Streets: Spanish in the Community* (SPAN M165XP) (Extended Data 2-1 for syllabus) and *Topics in Community Engagement: Latinos, Linguistics, and Literacy* (SPAN M172XP) (Extended Data 2-2 for syllabus), enrolled third and fourth year undergraduate students with bilingual or professional working proficiency in Spanish who were interested in engaging with local Hispanic-serving community partners. During the first week of class, students who expressed interest in working with Knowing Neurons to translate articles from English into Spanish were screened through a short translation exam. They were provided four paragraphs from a previously untranslated Knowing Neurons article and were asked to translate it over the course of 1 week. The course instructors then checked their translations for (1) grammar, (2) orthography, (3) lexics, (4) syntax, and (5) scientific accuracy. We accepted students whose sample translations showed a language proficiency ranging from B2 to C1 based on the Common European Framework of Reference for Languages (CEFR; Council of Europe, 2023) standards for lexical, grammatical, and orthographic competence.

10.1523/ENEURO.0392-23.2023.s2-1 Extended Data 2-1 **Course Syllabus for SPAN 165XP (Taking it to the Street: Spanish in the Community).** Here is the updated syllabus of the course offered in the spring of 2023 by the Department of Spanish and Portuguese at UCLA. Download Extended Data 2-1, DOC file.

10.1523/ENEURO.0392-23.2023.s2-2 Extended Data 2-2 **Course Syllabus for SPAN 172XP (Topics in Community Engagement: Latinos, Linguistics, and Literacy).** Here is the updated syllabus of the course offered in the spring of 2023 by the Department of Spanish and Portuguese at UCLA. Download Extended Data 2-2, DOC file.

More specifically, within this B2/C1 framework, a student had to demonstrate intelligible continuous writing, following standard layout and paragraphing conventions. Their spelling and punctuation had to be accurate or reasonably accurate allowing for some signs of mother tongue influence. Additionally, the student had to show a high degree of grammatical control and absence of mistakes that led to misunderstanding. Occasional “slips” or non-systematic errors and minor flaws in sentence structure were tolerated if they were rare and could be easily corrected. Last, lexical accuracy had to be generally high, though some confusion and incorrect word choice were permitted.

To maintain a high instructor-to-student ratio and guarantee a fast publication turnaround (no more than 2 weeks), we typically accept 2–5 undergraduate translators per academic quarter (10 weeks of instruction). To date, 25 undergraduate translators have participated in the program, translating 102 Knowing Neurons articles (Extended Data 2-3).

10.1523/ENEURO.0392-23.2023.s2-3 Extended Data 2-3 **Alphabetical list of articles translated between April of 2021 and June of 2023.** A total of 102 articles have been translated in a span of 7 academic quarters (spring 2021 – spring 2023; 26 months). A large range of topics (article titles), spanning multiple unique subject areas (“topic tags”, used to index content in the Knowing Neurons website) have been translated. All articles in their original English version can be found at https://knowingneurons.com, and all of the translated versions in Spanish can be found at https://knowingneurons.com/es. Download Extended Data 2-3, DOCX file.

10.1523/ENEURO.0392-23.2023.s2-4 Extended Data 2-4 **Lesson Plan Development Template.** Students are provided with this template to develop their lesson plans with the supervision of their professors in the Department of Spanish and Portuguese and Brain Research Institute. Download Extended Data 2-4, DOCX file.

### Translation process

Students who were deemed skilled enough were provided four full articles to translate over the course of a 10-week academic quarter, at a rate of one article every 2 weeks. The actual translation process was aided by the use of a professional translation management system (*PhraseTMS*, formerly *Memsource*) (Phrase, 2023), which students learned to use via video tutorials available from the vendor.

After each translation draft was submitted by the student translators, they were checked by (1) a Spanish linguistics graduate student reader and/or a Spanish linguistics professor for language usage and (2) by a neuroscience graduate student reader and/or neuroscience professor to validate its scientific accuracy. A 1 h meeting was subsequently held with the undergraduate translators to review linguistic errors or scientific inaccuracies in the translations before one final read-through of the draft to check for typos and to make any remaining minor edits. Final translated documents were received by Knowing Neurons for publication in the Spanish version of the platform at https://knowingneurons.com/es.

**Figure 2.**
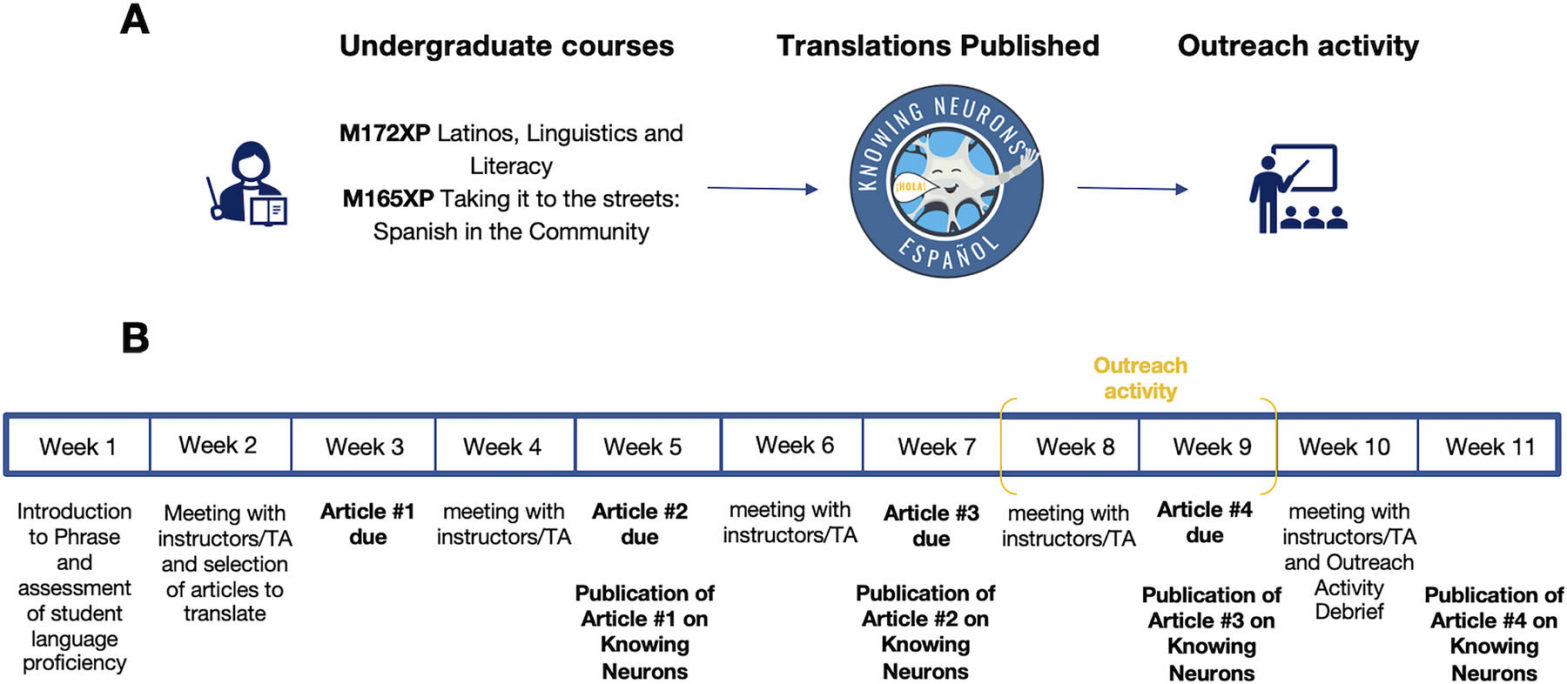
Outline of the translation process. ***A***, Overall, students enrolled in two undergraduate courses (see Extended Data 2-1 and 2-2 for the syllabi) in the Spanish and Portuguese Department at UCLA translate articles from English to Spanish, which are then published on the Spanish version of the Knowing Neurons platform: https://knowingneurons.com/es/ (see Extended Data 2-3 for the inaugural list of translations). To promote their work, students conduct an outreach activity locally in Los Angeles, California (United States). ***B***, Timeline of the translation process during a 10-week quarter (which includes an 11th week, corresponding to finals week). Students translate an article at the rate of one every 2 weeks, starting in week 2, and they conduct their community outreach activity between weeks 8 and 9 (see Extended Data 2-4 for the lesson plan template), to allow time during week 10 for a debrief. In between translation draft submissions, biweekly meetings with the students are scheduled to review the translation work, offer feedback, and suggest edits.

In parallel with the translations, the undergraduate students were also asked to put together an outreach activity to complement one of their translated articles. Example activities have included podcasts, interactive presentations, audible articles, or more recently, lesson plans (Table 2; Extended Data 2-4 for the lesson plan template). This provided students with a real-world opportunity to practice their Spanish and promote neuroscience, by incorporating the scientific elements of one of their translated articles into an activity implemented in a Spanish-speaking community center or high school classroom. The course instructors worked closely with the translators, not only to guide on language usage and scientific accuracy but also to identify reasonable student learning objectives as well as appropriate interactive lessons that effectively teach the science behind the translated article. For an outline of the whole process, see Figure 2.

**Table 2.** Summary of outreach activities associated with the Knowing Neurons article translations

Academic quarter	Outreach activity
Spring 2021	Podcast^a^: *Educación, carrera e identidad con Carlos Portera-Cailliau* (Education, Career, and Hispanic Identity with Carlos Portera-Cailliau). This podcast episode centers around a Spanish neuroscientist's journey into STEM, medicine, and academia. He provides candid advice and shares his experience transitioning to his current role from a Spanish-speaking background
	Presentation^b^: Students prepared a Google Slides presentation that outlined different career trajectories in the sciences. This included geology, oceanography, medicine, research, and more. Students also received a general introduction to “what is science?”. After reviewing the presentation on different scientific careers, students played a Kahoot, an interactive quiz-style game, to assess their engagement
Fall 2021	Presentation^c^: Students generated a presentation on epilepsy to a predominantly Spanish-speaking group of advanced placement Spanish and/or bilingual program students. Their primary aim was to reduce stigma surrounding epilepsy. Demographic information and attitudes toward the presentation were assessed via Google Form, and the data were presented to and discussed by the whole translation team
Winter 2021	Presentation^d^: Students conducted a presentation on science in general and the imposter syndrome to a predominantly Spanish-speaking group of advanced placement Spanish and/or bilingual program students. Demographic information and attitudes towards the presentation were assessed via Google Forms, and the data were presented to and discussed by the whole translation class
Spring 2022	One audible article^e^: Recording of the translated article: *¿Las personas con dyslexia leen y escriben al revés?* (Do People with Dyslexia Read and Write Backwards?). This provides an alternative method for individuals (including the sight impaired) to consume the Knowing Neurons content beyond reading the written articles.
	Presentation and fair^f^: Presentation about Knowing Neurons, neuroscience, and the state-of-art of representations of Latinx in STEM at a booth at a career fair. The activity also involved the giveaway of flyers providing information about the concepts presented as well as brain-related UCLA merchandise. The students also conducted a survey to evaluate the impact of the booth
	Podcast^g^: *Hormonas reproductivas y barreras educativas con Stephanie Correa* (Reproductive Hormones and Educational Barriers with Stephanie Correa). This podcast is a more technical episode about Dr. Correa's research, mainly focusing on how reproductive hormones regulate homeostasis and metabolism. She dives into some of her projects and elaborates on why she focuses on this area of research
Fall 2022	Outreach activity and lesson plan^h^: Students deconstructed the Knowing Neurons article: *El cerebro está más cerca de la tripa de lo que parece!* (Your Gut Is Closer to Your Brain Than It Appears!), on the gut microbiome and its relation to the brain article. The implemented lesson plan included an outreach activity that involved designing an experiment to investigate the role of different bacteria types on behavior in mice
Winter 2023	Outreach activity and lesson plan^b^: Students deconstructed the Knowing Neurons article: *¿Por qué nos hace sentir tanto la música?* (Why Does Music Make Us Feel So Much?), on the effects of music on the brain. The implemented lesson plan included an outreach activity that involved writing a song and identifying the anatomical regions involved in the brain's rewards system
	Outreach activity and lesson plan^i^: Students deconstructed the article: *Cuando tu cerebro usa Twitter: la neurociencia de las redes sociales* (This Is Your Brain on Twitter: The Neuroscience of Social Media), on how the brain feels reward on social media. The implemented lesson plan included an introduction to the brain's reward system and a Kahoot quiz
Spring 2023	Outreach activity and lesson plan^j^: Students deconstructed the Knowing Neurons article: *Entrenamiento con pesas y sus beneficios cognitivos: poniendo en forma a tu cerebro* (How Weight Lifting Gets the Brain in Shape), which discusses the cognitive benefits of exercising. The implemented lesson plan included an activity that involved identifying household items and spaces that could be used to exercise

Description of presentations, podcasts, audible articles, outreach activities, and lesson plans produced by undergraduate student translators between the springs of 2021 and 2023.

a https://open.spotify.com/episode/4wstIJ6MkxVVGACKudSzZd.

b Presented at Mar Vista Family Center, Culver City, California (United States).

c Presented at Herbert Hoover High School, Glendale, California (United States).

d Presented at San Pedro High School, San Pedro, California (United States).

e https://knowingneurons.com/es/blog/2023/07/10/dislexia-leen-y-escriben-al-reves/.

f Presented at Neuwirth Leadership Academy, Los Angeles, California (United States).

g https://open.spotify.com/episode/7kfKAAE7sHQbOL2h6MuQiz.

h Presented at Hawthorne High School, Hawthorne, California (United States).

i Presented at Academia Advance Charter School, Los Angeles, California (United States).

j Presented at Phineas Banning High School, Los Angeles, California (United States).

The number of activities produced, and the number of students assigned per activity (either two or three), depends on the number of translators recruited each academic quarter and the complexity of the activity. Podcasts are available on Spotify (links in Table 2), and lesson plans in Spanish are specifically archived at https://knowingneurons.com/es/planes-de-leccion/.

### Code accessibility

The code/software used to analyze the data presented here is freely available online at https://github.com/albaperis/Translation-Project-Knowing-Neurons. The code is available as an Extended Data.

## Results

Since the inception of this project in the spring of 2021, the following Spanish language content has been created: 102 article translations (Extended Data 2-3), 2 podcast interviews with Spanish-speaking neuroscientists, and 1 Audible article. Additionally, several outreach activities were designed and implemented, namely, four lesson plans and four presentations at local bilingual high schools or community/family centers (Table 2). In the early stages of the project, the Spanish language translations were posted on the main Knowing Neurons webpage (https://knowingneurons.com) under the “languages” tab. However, with an ever-expanding list of articles, in January of 2023, we generated a sister Spanish language website (https://knowingneurons.com/es/) where all the Spanish language articles were ultimately migrated.

**Figure 3.**
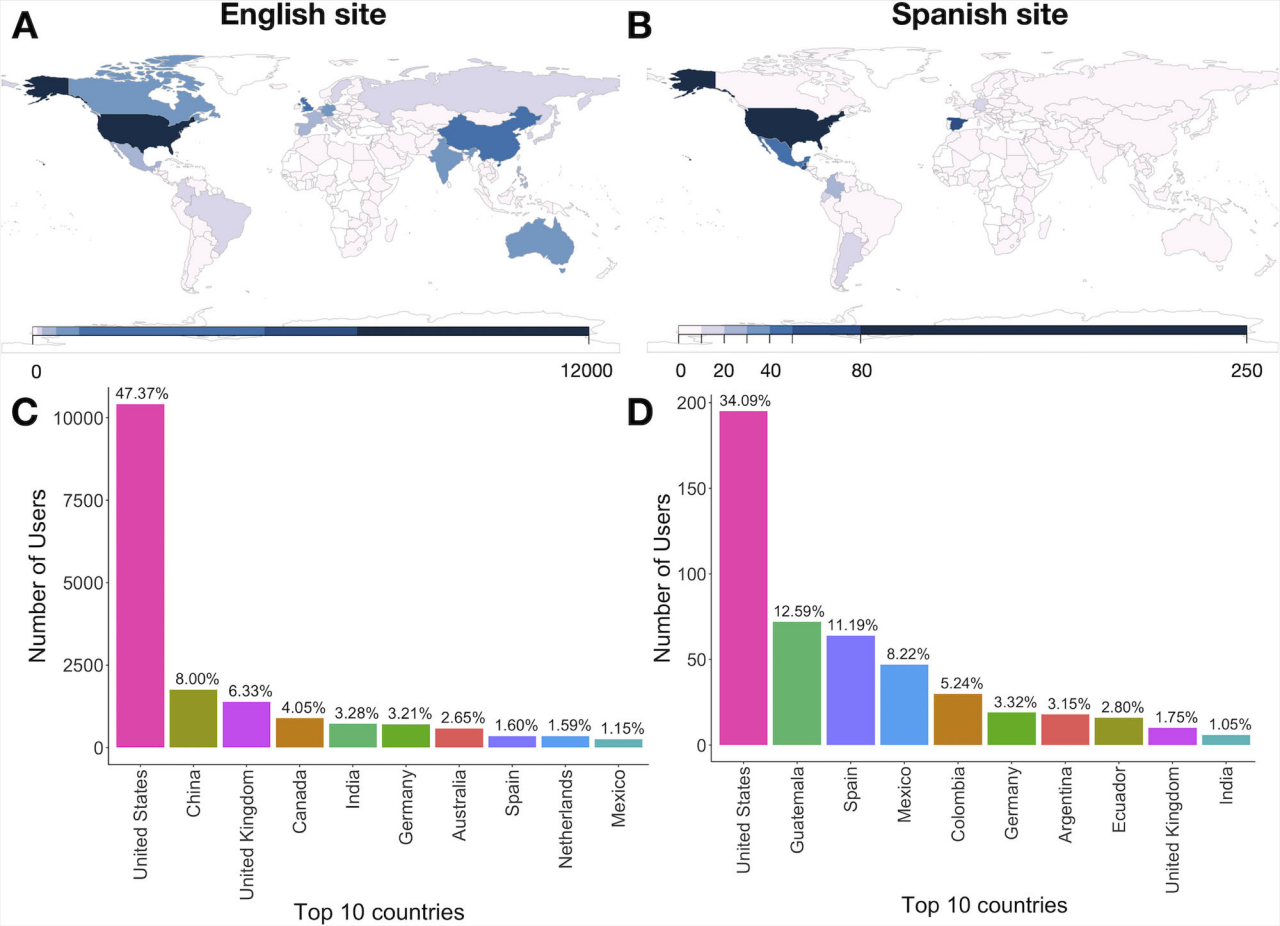
World map of visitors to the Knowing Neurons Spanish and English domains from January 1 to June 30, 2023. The top panels show world maps of readership in the English (***A***) and Spanish (***B***) sites. The shaded scales represent the number of times the sites were accessed by a user. The bottom panels indicate the top 10 countries accessing the English (***C***) and Spanish (***D***) sites. Percentages of the total number of site visits are shown above the bar plots. The top 10 countries visiting the English language site represent 79.23% of all users, while the top 10 countries visiting the Spanish language site represent 83.4% of all users.

In an initial attempt to assess the impact and potential of this translation initiative, we first compared the number of website users to the English and Spanish Knowing Neurons websites in the first 6 months of 2023, since the official launch of the Spanish language domain. This would give us a glimpse of the initial interest in the new Spanish language site compared to the baseline user activity of the well-established English language site and provide us with a rough standard of what user engagement we can ultimately aim for. At first glance, there is a large difference in visitor volume to both sites, with 10,000+ visits to the English site as opposed to 300+ visits to the Spanish one (Fig. 3), but this is expected given that the flagship Knowing Neurons English website has 5 times more content and has been active since 2012 while the slimmer Spanish site was only launched in 2023. Indeed, while the English site already has an established number of visitors, the Spanish site is only now being advertised.

A closer inspection of the geographical origin of the site visits reveals that as expected, most of the activity to the English language website originates from countries where English is an/the official (or de facto) language, such as the United States, the United Kingdom, Canada, India, or Australia (Fig. 3*A*,*C*). However, other countries such as China, Germany, Spain, the Netherlands, and Mexico also appear in the top 10 list, suggesting that perhaps bilingual populations are accessing the content as well. In contrast, the majority of visits to the Spanish language website arise from countries where Spanish is either an/the official language, such as Guatemala, Spain, Mexico, Colombia, Argentina, or Ecuador, or where there is a large Spanish-speaking population, such as the United States (Fig. 3*B*,*D*). This confirms that most traffic to the Spanish language website originates from countries/populations that speak the language, indicating that although low, the Spanish content readership is likely the result of purposeful website activity rather than random or off-target domain access. Indeed, the total (worldwide) average time spent in the Spanish language domain is 8 min and 58 s (Table 3), suggesting that the articles are being read, assuming an average silent reading rate of 175–300 words/minute (Brysbaert, 2019) and that the average Knowing Neurons article has 500–1,500 words. Interestingly, the total (worldwide) average time spent on the English site was much lower (1 min and 14 s), likely representing a combination of article reading as well as quick site browsing by returning users looking for new material. Notably, Germany, the United Kingdom, and India also appear in the top 10 countries accessing the Spanish language site (Fig. 3*D*), which could be due to bilingual readers accessing the content or due to the presence of Knowing Neurons members in those countries working to help launch the novel website.

**Table 3.** Worldwide user data for the knowing neurons English and Spanish language websites between January 2023 and June 2023

Country	Users	New users	Avg. session duration
English language site			
Total (worldwide)	21,969	21,729	00:01:14
United States	10,407	10,304	00:01:19
China	1,757	1,755	00:00:15
United Kingdom	1,391	1,372	00:00:55
Canada	889	881	00:00:57
India	720	717	00:01:09
Germany	705	691	00:01:16
Australia	583	578	00:00:34
Spain	351	339	00:01:04
Netherlands	350	345	00:00:52
Mexico	252	247	00:01:06
Spanish Language Site			
Total (worldwide)	572	495	00:08:58
United States	195	145	00:12:14
Guatemala	72	70	00:01:27
Spain	64	60	00:03:03
Mexico	47	44	00:01:17
Colombia	30	29	00:04:14
Germany	19	15	00:08:17
Argentina	18	17	00:03:47
Ecuador	16	16	00:02:20
United Kingdom	10	6	00:07:57
India	6	5	00:06:55

Column 1 lists the top 10 countries by usership for both the English (top) and Spanish (bottom) language websites. Total (worldwide) refers to usership data stemming from every country, not just the top 10 listed. Column 2 presents the total number of users accessing the English or Spanish language websites, while column 3 solely represents new users. “User” is defined as an individual who has interacted with one of the Knowing Neurons websites. “New User” is defined as a person who has visited one of the Knowing Neurons websites for the first time. Column 4 summarizes the average session duration, or the average length of time (in hours, minutes, and seconds) that users have spent on either of the Knowing Neurons websites. All data were obtained through Google Analytics.

**Figure 4.**
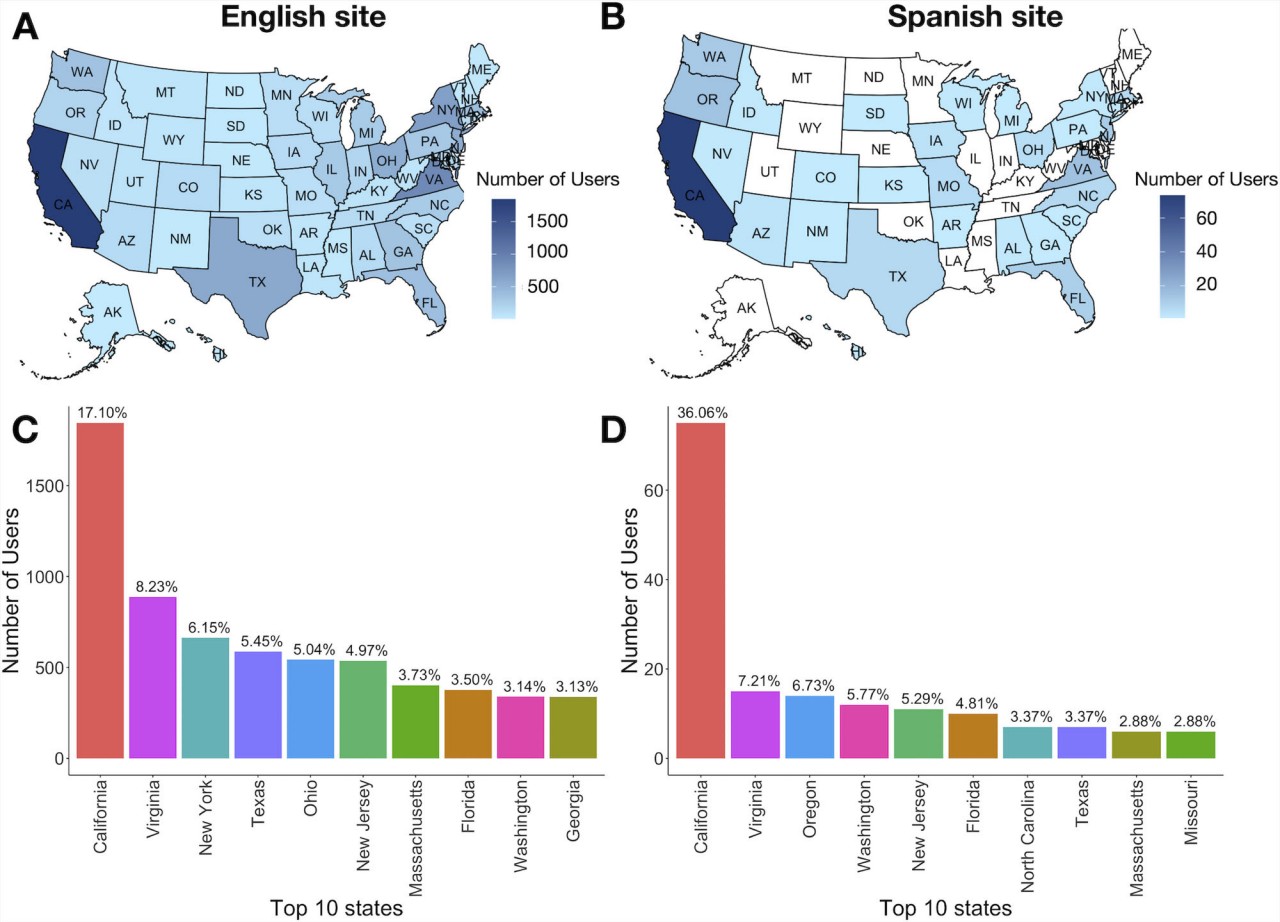
Number of users to the Knowing Neurons Spanish and English domains from January 1 to June 30, 2023 based in the United States. The top panels show US maps representing the number of users from each state accessing the English (***A***) and Spanish (***B***) sites. The shaded scales represent the number of times the sites were accessed by a user. The bottom panels show the top 10 states accessing the English (***C***) and Spanish (***D***) sites. Percentages of the total number of site visits are shown above the bar plots. The top 10 states visiting the English language site represent 60.44% of all users, while the top 10 states visiting the Spanish language site represent 78.37% of all users.

Given that most readership of the Knowing Neurons material originates in the United States, we similarly compared the user activity here between the English and Spanish websites. Readership to the English language domain is spread throughout the country, albeit unevenly, with a notable concentration in California, the birthplace and permanent headquarters of Knowing Neurons (Fig. 4*A*,*C*). Readership to the Spanish language domain is much more sparse but also centered in California (Fig. 4*B*,*D*). Time spent on the English site averaged 1 min 19 s (Table 4), but as with the global visitor data, the reduced amount of time is unsurprising given the much larger number of site visitors. It is expected that some of that activity is due to established visitors performing quick searches, which will necessarily lower the overall average of time spent on the site. Conversely and coincident with the worldwide user data, the average session duration of readers within the United States was higher, at 12 min and 14 s for the Spanish website. Again, although the number of users is very low, this would suggest that there is a lengthy and meaningful interaction with the novel website by interested users, with the exception of Texas, which has an unusually low average session duration of just 7 s. We also note that the comparatively lengthy average session duration for the Spanish website might in part be explained by higher numbers of Spanish language learners and heritage speakers accessing the articles. They would understandably interact with the material more slowly, and we expect these readers to be a sizeable portion of the website users as our outreach efforts are mostly targeting K–12 students.

**Table 4.** User data for the knowing neurons English and Spanish language websites between January 2023 and June 2023 within the United States

Region	Users	New users	Avg. session duration
English language site			
Total (50 states)	10,782	10,304	00:01:19
California	1,844	1,782	00:02:08
Virginia	887	857	00:00:51
New York	663	639	00:01:17
Texas	588	565	00:01:17
Ohio	543	520	00:01:06
New Jersey	536	533	00:00:54
Massachusetts	402	388	00:01:17
Florida	377	348	00:01:14
Washington	339	305	00:01:00
Georgia	338	325	00:01:11
Spanish Language Site			
Total (50 states)	208	145	00:12:14
California	75	42	00:15:53
Virginia	15	13	00:09:04
Oregon	14	11	00:09:31
Washington	12	9	00:03:34
New Jersey	11	9	00:05:53
Florida	10	8	00:04:55
Texas	7	6	00:00:07
North Carolina	7	5	00:08:29
Massachusetts	6	3	00:18:15
Missouri	6	3	00:14:06

Column 1 lists the top 10 states by usership for both the English (top) and Spanish (bottom) language websites. Total (50 states) refers to usership data stemming from the whole country. Column 2 presents the total number of users accessing the English or Spanish language websites, while column 3 solely represents new users. “User” is defined as an individual who has interacted with one of the Knowing Neurons websites. “New User” is defined as a person who has visited one of the Knowing Neurons websites for the first time. Column 4 summarizes the average session duration, or the average length of time (in hours, minutes, and seconds) that users have spent on either of the Knowing Neurons websites. All data were obtained through Google Analytics.

## Discussion

Here, we describe a novel partnership between the Department of Spanish and Portuguese and the Brain Research Institute at UCLA, with the nonprofit neuroscience outreach organization, Knowing Neurons, to translate neuroscience content from English to Spanish. To our knowledge, such a collaboration to systematically translate a high-impact neuroscience portal into a second language has not been previously described. As part of this educational partnership, bilingual undergraduate students from the Department of Spanish and Portuguese undertake the translation of previously published neuroscience outreach articles under the mentorship of a linguist and a neuroscientist. The translators also conduct outreach activities in local high schools or community centers in the greater Los Angeles area to promote the translated content. In essence, our translation efforts have made extensive use of the resources and culture available at a research university, namely, bilingual undergraduate students enrolled in rigorous community service courses, bilingual graduate student readers and faculty, and partnerships with local community stakeholders.

In the present paper, we also provide a clear outline of how to successfully establish a partnership between the sciences and humanities through a translation initiative at a higher education institution. While there have been prior attempts to bridge the gap between the sciences and the humanities and the need for discussions and partnerships across these different disciplines have been acknowledged (Ross, 2016), they are nevertheless rare, particularly in higher education institutions, and in the context of scientific communication. Indeed, the lack of communication between the sciences and the humanities, or *The Two Cultures*, as described by Snow (1959) has long been an institutional issue (Snow, 1959; Meneghini and Packer, 2007).

Although it is too early to establish the long-term impact of this initiative, preliminary results suggest that there is a burgeoning interest in Knowing Neurons content throughout the Spanish-speaking world. This is evidenced by the list of top 10 countries visiting the Spanish language Knowing Neurons website containing Spanish-speaking countries in North, Central, and South America, as well as the Iberian Peninsula (Fig 3*B*,*D*). We acknowledge that the site visit numbers are ∼40 times lower than those of the English language website for the same time period, but lack of visibility is likely the main cause. Currently, the promotion of the Spanish language content has been limited to Los Angeles, California (United States), through the use of the outreach activities produced, compiled, and presented by the undergraduate student translators. Indeed, this is probably why over a third of the visits to the Spanish language site within the United States originate from California (Fig. 4*D*), compared to just one-sixth for the English language site (Fig. 4*C*).

In order to increase user activity with the Spanish language site, we are currently implementing a number of strategies. First, we are in the process of identifying bilingual schools and/or classrooms throughout the United States and beyond to promote the use of the lesson plans. Second, we are looking into recruiting additional Spanish-speaking neuroscience graduate students across the United States and throughout the Spanish-speaking world to increase the visibility of the Spanish language articles on a national and global scale. Last, we are generating a strong social media presence in Spanish-speaking circles. In fact, Knowing Neurons has already started to create accounts with relevant online media outlets like LinkedIn (https://www.linkedin.com/company/knowingneurons/), Instagram (@knowingneurons) and Twitter (@KnowingNeurons) to advertise and share the new bilingual content, as well as to stimulate interest in the Spanish language Knowing Neurons webpage as a reputable portal to learn about new developments in neuroscience.

To further inform us on how best to promote our new Spanish language site, we will better elucidate the readership profile of our website users in both our Spanish and English platforms, by continuing to implement user-tracking add-ons, such as Google Analytics. We hope that this will help us disentangle the English and Spanish language user profiles more effectively, particularly within the United States, a bilingual country where the majority of our readership comes from. We are particularly interested in learning how many users are bilingual and access articles in both English and Spanish as well as understanding who our English and Spanish audiences are and how they are interacting with our resources.

We have described that part of the translation initiative requires our students to produce different types of activities and/or materials that are employed in bilingual high school classrooms or community centers. This serves a few purposes, both at the academic and community level. First, it benefits our undergraduate student translators, as the implementation of outreach activities in Spanish not only allows them to practice their second-language skills but can help them develop transferable soft skills, such as public speaking, science communication, and teamwork, as they prepare to enter the workforce (Stevens, 2011; Yawson et al., 2016). Furthermore, it provides useful experience in communicating scientific findings to the general public for those who will continue their training as future researchers (Brownell et al., 2013). Second, faculty looking to integrate outreach components into their curricula can use our model as a guide. Scientific outreach practices are difficult to incorporate by researchers and scientists, particularly at an institutional level, as they are often undervalued or unrecognized in faculty reviews (Cameron and McNerney, 2006; Illes et al., 2010; Stevens, 2011) and can even negatively impact early career researchers in the eyes of more senior faculty (Filling the void, 2009; Illes et al., 2010). However, some of these impacts can be mitigated if the outreach is incorporated into a preexisting classroom curriculum (Stevens, 2011), as we present here.

Last, the creation of didactic content in Spanish allows for the promotion of neuroscience concepts to previously neglected populations, particularly non-English speakers locally (within the United States) and abroad (Silva et al., 2022). Importantly, the outreach materials produced are adapted to school children, and reaching younger audiences has been shown to increase their interest and understanding of science (Colón-Rodríguez et al., 2019). This is particularly critical given the underrepresentation of Hispanic graduates in STEM majors and careers. Specifically in the United States, only 12% of students graduating with a STEM degree are Hispanic (Fry et al., 2021) despite making up 20% of the college student population (Mora, 2022). Likewise, while Hispanic or Latino workers represent 18% of the US workforce, in STEM careers, they only make up 8% of the workers with a bachelor's degree or higher (National Science Board, 2021).

Our translation initiative can not only stimulate interest in neuroscience in a historically underrepresented group in higher education but can also widen the availability of neuroscience information to a larger global audience. Moreover, it has allowed us to generate guidelines that can be applied to the translation of these same articles into other languages (something that we are currently planning), or more broadly, implemented for translation work in other academic spheres. Given its potential impact and relatively low cost, we plan to continue translating new articles and to produce fresh didactic material on an ongoing basis. Importantly, as we curate the Spanish-language Knowing Neurons website, we will foster the same reputation as a reliable source for accessible educational materials and information on the nervous system that is currently enjoyed by the fully established and award-winning English language version of Knowing Neurons. Ultimately, we hope that our sustained efforts to produce and promote neuroscience content in Spanish will inspire neuroscience faculty, graduate, and undergraduate students in other communities to use the framework presented here to translate other neuroscience content into more languages. Achieving this goal will further expand global access to neuroscience educational material and research to everyone, making it truly universal.

## Synthesis

Reviewing Editor: Christine Portfors, Washington State University Decisions are customarily a result of the Reviewing Editor and the peer reviewers coming together and discussing their recommendations until a consensus is reached. When revisions are invited, a fact-based synthesis statement explaining their decision and outlining what is needed to prepare a revision will be listed below. The following reviewer(s) agreed to reveal their identity: NONE.

This is an interesting description of the process of creating Spanish access for Neuroscience outreach materials. The article is well written and will be beneficial for other faculty, departments or colleges interested in increasing accessibility of outreach materials.

There are only minor comments to address:

Figure 1 - the text suggests that Figure 1 will display the mission and purpose of the Knowing Neurons forum but figure 1 itself shows the structure of the organization and not what it does. The purpose of Figure 1 should be clarified and the text and figure revised appropriately.

Table 1 - there are actually 26 institutions listed, not 25 as indicated in the text.

Article selection - line 125 - how was "interest" determined? Was this based on the authors' interest or some other metric? Please clarify.

Table 2 - I am not entirely convinced that showing all 102 articles that have been translated to date is necessary. I imagine this list will be outdated by the time this article is published, and it makes for an excessively long table. Perhaps this would be better as extended data.

Line 203 - there is a typo in the link so it does not work

## Author Response

Synthesis Statement for Author (Required):

This is an interesting description of the process of creating Spanish access for Neuroscience outreach materials. The article is well written and will be beneficial for other faculty, departments or colleges interested in increasing accessibility of outreach materials.

We thank the reviewers for the positive comments and suggestions to improve the paper. We address each one individually below.

All text modifications on the accompanying manuscript are marked in either yellow highlight (new text), or red strikethrough (obsolete text).

There are only minor comments to address:

Figure 1 - the text suggests that Figure 1 will display the mission and purpose of the Knowing Neurons forum but figure 1 itself shows the structure of the organization and not what it does. The purpose of

Figure 1 should be clarified and the text and figure revised appropriately.

Figure 1 is meant to show the organizational structure of Knowing Neurons. However, in its present form it is indeed incongruent with the text, which solely mentions Knowing Neurons' mission. To address this issue, we have restructured the text (lines 100-107) to clearly describe both the structure and mission of Knowing Neurons. No changes to Figure 1 were necessary.

Table 1 - there are actually 26 institutions listed, not 25 as indicated in the text.

This oversight has been addressed in the text (line 109).

Article selection - line 125 - how was "interest" determined? Was this based on the authors' interest or some other metric? Please clarify.

We acknowledge that "order of interest" suggests some form of classification scheme, which requires explanation. In actuality, our "interest" was based on pedagogical relevance, with topics considered more basic/introductory given priority. We have made the relevant changes to the text (lines 128-132) to clarity how our papers were ranked for translation.

Table 2 - I am not entirely convinced that showing all 102 articles that have been translated to date is necessary. I imagine this list will be outdated by the time this article is published, and it makes for an excessively long table. Perhaps this would be better as extended data.

We agree that our list of articles makes for an unusually long and unwieldy table. Likewise, we agree that the list will be outdated soon, as we are expecting another dozen translations by the end of the year alone. That said, our intention was simply to catalog the papers whose translation allowed the launch of the Spanish-language Knowing Neurons website, which is why we decided to include this table in the first place. As suggested by the reviewers, a good compromise is to keep the information in the paper, but as extended data. We have therefore reclassified "Table 2" as "Extended data 2-3". We have similarly renumbered the other Tables and Extended data files as needed.

These changes are visible in lines 137, 142, 144, 166, 186, 195, 205, 206, 208, 237, 252, 440, 442, 443 & 447.

We also added the required Extended Data Legends (lines 470-489).

Line 203 - there is a typo in the link so it does not work

The typo has been addressed in the text (line 210).

Additional change to text for added clarity:

While waiting for the review process, we calculated the number of UCLA students that enroll in all of the Spanish language courses offered by the Department of Spanish and Portuguese. The text was slightly modified to include this number (lines 118-119; green highlight). We felt that it helps readers establish the impact the Department has on our student population.
