# Supplementary material for: Increasing Accessibility to Neuroscience through Translation: Going beyond the English Language
Source: eNeuro. 2024 Jan 10;11(1):ENEURO.0392-23.2023. doi: 10.1523/ENEURO.0392-23.2023 (PMC11078105; doi:10.1523/ENEURO.0392-23.2023)
Supplement: Extended Data 2-1 — Course Syllabus for SPAN 165XP (Taking it to the Street: Spanish in the Community). Here is the updated syllabus of the course offered in the spring of 2023 by the Department of Spanish and Portuguese at UCLA. Download Extended Data 2-1, DOC file. [file eneuro-11-ENEURO.0392-23.2023-s001.doc]

SPAN 165XP/167XP
Taking it to the Street: Spanish in Community University of California, Los Angeles Department of Spanish and Portuguese

Spring 2023 Thursdays 2:00 to 4:50pm
Haines A74 5 units


The Department of Spanish and Portuguese at UCLA acknowledges the Gabrielino/Tongva peoples as the traditional land caretakers of Tovaangar (the Los Angeles basin and So. Channel Islands). As a land grant institution, we pay our respects to the Honuukvetam (Ancestors), ‘Ahiihirom (Elders) and ‘Eyoohiinkem (our relatives/relations) past, present and emerging.
Email: carla.suhr@ucla.edu
Instructor: Dr. Carla Suhr
Office: Rolfe Hall 5326
Office Hours: T 11:00am to 12:00pm and 5:00pm to 6:00pm and by appointment In- person or by Zoom: Meeting ID: 956 9373 8665 Passcode: 575744
Course	website	URL:	https://bruinlearn.ucla.edu/courses/164436

Prerequisites:
Junior Standing or above (Spanish, Culture, and Community and Chicano/a Studies).
SPAN 25 or the equivalent.
In order to engage in this course successfully, you need to have already attained a strong level of written and spoken Spanish.

Course Description:

Service Learning is a teaching and learning strategy that integrates meaningful community service with instruction and reflection to enrich the learning experience, teach civic responsibility, and strengthen communities. This is a service learning course that specifically allows you to use the cultural and linguistic knowledge you have acquired in your Spanish classes in a real-world setting through 6-8 hours per week of service in the Latinx community.

Class time will be dedicated to lectures through which you will be introduced to social topics, in connection to the Spanish-speaking community of the United States, and follow-up communicative activities in Spanish. You should come to class prepared to participate and to enthusiastically engage in a variety of activities in groups and pairs. In class, your instructor will provide opportunities to listen, speak, read, and write in Spanish.

This course seeks to:

Help you make connections between the discipline (Spanish) and the real world
Provide you with an expanded vision of the culture and needs of our local and global Hispanic communities
Provide you with purposeful skills that can be useful in your future career
Encourage civic engagement and promote the empowering notion that each student can make a difference
Help you improve or further develop your skills in spoken and written Spanish
Acquaint students with the concept of Community Service Learning as a valuable form of pedagogy

Course Learning Objectives:

By the end of this course, you will be able to:

Explain comprehensively at least 5 social issues that disproportionately affect the Spanish- speaking communities in California in Spanish
List and describe at least 10 initiatives advocating for social justice and non-profit organizations providing medical, housing-assistance, legal, social, and educational services to the local Latinx communities
Provide examples on how purposeful skills in problem solving and interpersonal communication that you developed through the quarter helped you perform assigned tasks successfully at your non-profit organization and how you expect them to be valuable in your future career
Communicate verbally and in written format at a B2-C1 Spanish level both in class and in your service site with peers from Spanish speaking and Latinx cultures (see complete level description on the course website).
Understand a wide range of demanding, longer texts, and recognize implicit meaning.
Express yourself fluently and spontaneously without much obvious searching for expressions.
Use language flexibly and effectively for social, academic and professional purposes.
Produce clear, well-structured, detailed text on complex subjects, showing controlled use of organizational patterns, connectors and cohesive devices.
Create, from planning to execution, at least 1 useful product (resource) for your service site based on a community-identified need by making use of the cultural and linguistic knowledge you have acquired in your Spanish classes that will contribute to social justice efforts
Distinguish between Critical Community-Engaged Learning and other Community Service Learning models and explain what makes Community Service Learning a valuable form of pedagogy

Required Texts: All readings and homework will be posted to the course website. These materials have been specifically selected to best support your service learning in this course.
Note: Immersive Reader is a feature, located along the top right of most Bruin Learn content pages, that assists a variety of learners by:
reading text aloud at adjustable speeds
removing visual clutter to minimize distraction
allowing users to adjust fonts and sizes (especially helpful for dyslexic and low-vision users)
providing adjustable spacing to help support reading comprehension and retention

Immersive Reader also facilitates language translation and includes a picture dictionary, which can assist students with communication difficulties.

The Immersive Reader tool aligns with principles of Universal Design for Learning (UDL) by fostering multiple means of representing information and multiple ways for learners to engage with that information.

Note about technology: Browsers: You will need to use a browser as your primary method of accessing the course website. The Canvas mobile apps should only be used as a supplement. To learn about browser compatibility with Canvas, visit the Supported Browsers. Our Canvas course site is your home base, with links to everything you need. Make sure that your campus e-mail account and access to Canvas are functioning and check your e-mail regularly for updates and announcements. In the classroom, you may use your laptop for class-related purposes only. Unless you choose to do your service virtually, you will not need a laptop for your service.
Course requirement:

Service Learning: Students enrolled in SPAN 165XP will identify an appropriate placement site, with the help of the instructor. You are required to perform a minimum of 6-8 hours per week for a total of 60-80 hrs of unpaid service to the organization they choose, over the course of the quarter.
These hrs will be spent as follows:
50 hrs for the assigned service (to be signed in the timesheet by the supervisor of your service site) 10-30 hrs for the final project and transportation
Preparation for your service: A week before the course starts, you will be provided (via email and through the course website) with: 1) A list of all our community partners for this course. Please revise the description, the scope of work of each one carefully! Most sites offer in-person volunteering but a few of them have the option of volunteering in a hybrid format or completely virtually. 2) A form for you to fill out with your top three preferred organizations (please fill it out by the due date). When choosing a site, keep in mind that it should be a site working on a social topic that you are passionate about and where you can meet your learning objectives as well as the course objectives. The site you choose will offer you the opportunity to use your Spanish actively. Your primary activity at the service learning site should involve some combination of reading, writing, speaking, or translating Spanish, at least 80% of the time. You will be introduced to the supervisor of one of those organizations for you to coordinate an interview and the orientation. It is very important that you are proactive at this time. Some organizations take up to 2-3 weeks to get a student volunteer started, especially those that require TB tests and background checks. You must contact the supervisor of your chosen site immediately; if you do not hear back from the supervisor within 1-2 days, contact the supervisor there and your professor. It is imperative that you begin your field work by WEEK 2 of the academic quarter, at the very latest. It is preferable to begin during Week 1 if possible. You must have your site supervisor sign your time sheet at the end of the quarter. If you miss any of your regular service hours, you must make them up. For every 4 hours missed, your final grade will be lowered by one full letter grade. If you anticipate needing more time to finish your hours, please ask for an extension. The latest submission may be June 20th.
Feel free to discuss any concerns you may have with the instructor at any point. If it appears that, after having made a good-faith effort, you are unable to meet your learning objectives and the course objectives at the placement site you have chosen, you may select another placement site to switch to. It is better to switch as soon as possible rather than spend an entire quarter in a place that is not meeting your needs/objectives. However, please be sure to communicate openly with everyone involved, and never leave people guessing. Organizations come to depend on their volunteers, and will need to make alternate

arrangements if you plan to stop your service there. Signed timesheet is due on June 11th. Requested extension up to June 20th.

Advice for Doing Well
Come to class and to your service site every week and engage in Spanish. Challenge yourself!
Take notes: write down new things, but also your own thoughts and impressions. Always try to write things in your own words (or images!). This will help you especially with your journal writing.
Stay present and listen to others actively. This requires you to stay away from using technology for non-class-related purposes. You are a valuable member of our class community and we need you present in order for it to thrive!
Review the course material daily, even if just for a bit. Refresh your memory right before class.
Come see me in office hours! I am always happy to answer questions or review your presentations in my office hours.
Evaluation Criteria:
In this class, you will spend 60-80 hours “in the field”, actively engaged in the learning process as well as attend and participate in several on-campus class meetings that involve active participation and oral presentations. Your grade will be determined by how well you can demonstrate what you have learned as a result of your service activity and seminars, including your ability to express yourself (orally and in writing) in Spanish.
Participation in class meetings and assignments 25% Reflection Journals 25%
Mid-term Oral Presentation 25%
Final Presentation (Service and Group Project) 25%


Course Grade
The final course grade will be based on a percentage system of the points accumulated during the quarter, according to the following scale:
100-98 = A+
97-94 = A
93-90 = A-
89-87 = B+
86-83 = B
82-80 = B-
79-77 = C+
76-73 = C
72-70 = C-

69-67 = D+
66-63 = D
62-60 = D
59-0 = F
For P/NP grading (undergraduates), P = C (73) or better. For S/U grading (graduate students), S = B (83) or better

Descriptions of Assignments:
Note: Assignment rubrics and examples of exemplary assignments will be provided to you via the course website.
Participation and assignments: On-campus meetings will involve active participation and oral presentations in Spanish. You should come to class prepared to participate in, to contribute to class discussions and to enthusiastically engage in a variety of activities in groups and pairs. In class, your instructor will provide opportunities to listen, speak, read, and write in Spanish. All the class PPT presentations and materials will be uploaded on the course website. However, without attending class, students will be unable to contribute to class learning and participation. If you are unable to attend class, you may schedule an in-person or Zoom appointment with your instructor in order to make up for your participation and ask any questions you may have. There will be a total of 5 written complete/incomplete assignments that you must submit via the course website. You will also have the opportunity to participate in a discussion based on the assignments during class time. Your instructor will evaluate your interpersonal communication (speaking and writing) and will provide you with feedback throughout the course.

Reflection Journals: You will write 3 journal entries. Journal 1 and 2 entries should be 1-2 pages in length in Spanish. You should write your journal entries as soon as possible after you leave your service learning site, while the material is still “fresh”. Remember that your journal should not simply be an informal record of your impressions, but rather a critical analysis of activities and dynamics at your site. Your instructor will provide you with questions and topics you should address in your journal entries. The final journal will be a description and analysis of the service-learning experience and what you have learned through your own service, seminars, and your interaction with the other students in the course. This is a reflective essay in which you must cite a minimum of three sources and include a bibliography. This final reflective essay is to be 3-5 pages in length, typed and double-spaced, must be written in Spanish, and must include a bibliography. Your instructor will provide you with specific instructions and criteria for evaluation at the appropriate time during the quarter. The final journal is due no later than June 11th. (Early submissions are encouraged). Your journal entries must be typed and doubled-spaced and must be submitted via the course website (journals 1 and 2) and via email (journal 3) (by 11:59PM on the due date)

Mid-term Oral Presentation: Students will make a 10-15 minute oral presentation during Week 4 and 5 to the class describing your service site experience to that point. All the points that must be included in your presentation will be posted on the modules section of the course website in the Week 5 module. The oral presentation will be entirely in Spanish. You may use note cards but you must not read your presentation. Please note: you may present within the time limit for your presentation. You may be present for all of the oral presentations to provide feedback to your classmates.

Group Project and Final Oral Presentation: You will make a final project (in Spanish) with the students working at the same site as you. This project will be a product from which the organizations could benefit

and make use of in the future (1hr workshop, 5 min educational video, field research, etc). At least 10 of the 60 hrs of the service will be used to work with your group on the final project. It is highly encouraged that students submit this project to participate in the Dolores Huerta Community Service Award and the Undergraduate Research Week. The details for each assignment will be determined by the coordinator of your site in collaboration with the students once the participants for each site have been confirmed. The final project outline is due on May 11th (Week 6). The draft for the final group project is due on June 1st (Week 9). It will be presented on June 8th (Week 10, in groups).
Each group will present their final project in 15 mins. Your presentation must be accompanied by a PowerPoint or poster that includes materials that help the other students visualize the work that you have done and what you have learned and gained from the experience. You will receive specific instructions and criteria for evaluation from the instructor prior to the oral presentation. These instructions will be posted in the modules section of the course website on the Week 10 module. Please note: You may use note cards but you must not read your presentation. you may present within the time limit for your presentation. You must be present for all of the oral presentations to provide feedback to your classmates. You must present as scheduled. There will be no make-up oral presentations.

Course Schedule:


Date	Topic	Seminar Learning Objectives	Service and Homework (due
the week it is listed)	
Week 1	Presentación del curso e introducción al aprendizaje a través del servicio y a las organizaciones sin ánimo de lucro	Conocer todos los aspectos del curso incluyendo objetivos, agenda, criterios de evaluación y acomodaciones
Familiarizarse con los conceptos y modelos relacionados con el aprendizaje a través del servicio y las mejores prácticas de trabajo comunitario
Filtrar las organizaciones sin fines de lucro disponibles de acuerdo a los intereses propios y establecer contacto con la organización para iniciar el voluntariado
Distinguir entre los modelos de aprendizaje a través del servicio tradicional y crítico	Preséntese a sus compañero/as
Preferencia de voluntariado	
Week 2	Aprendizaje a través del servicio en español
Hispanohablantes en EEUU	Familiarizarse con el vocabulario relacionado con el servicio en español Revisar las lecturas y aprender de diferentes conceptos y modelos del aprendizaje a través del servicio
Aprender acerca de los datos demográficos de los latino y el español en EEUU Reflexionar sobre el concepto de actitud lingüística
Practicar las destrezas orales de español en un debate en el que se reflexiona sobre la actitud lingüística de las variantes del
español en EEUU	Service 6-8 hours
Assignment 1: Construyendo Puentes" Due
Assignment 2: "Traditional vs Critical Service Learning" Due
Journal 1 Due	

				
Week 3	La población latina en Los Ángeles. Reflexión sobre las disparidades. Iniciativas (agentes sociales de concienciación, empoderamiento, manifestaciones y revueltas, reformas políticas, organizaciones de apoyo)	Aprender sobre los datos demográficos de la comunidad hispana en el condado de Los Ángeles
Identificar las diferentes respuestas que surgen ante las disparidades en el ámbito de activismo social, artístico y de reformas
Aprender sobre la labor por una educación digna como elemento dinamizador esencial del levantamiento social
Revisar directrices y estrategias para las presentaciones orales	Service 6-8 hours	
Week 4	Alfabetización de medios. Águilas del desierto.
Estereotipos e identidad.	Reflexionar sobre la realidad del desierto fronterizo y Conocer la labor que realiza Águilas de desierto
Aprender sobre conceptos conectados a la identidad	Service 6-8 hours
Assignment 3 "Águilas del desierto. Los migrantes muertos del desierto de Arizona" Due	
Week 5	Midterm Oral Presentations	Desarrollar destrezas orales y auditivas a nivel avanzado en español Familiarizarse con las organizaciones sin
fines de lucro que proveen servicios para la comunidad latina a través de las presentaciones de sus compañero/as	Service 6-8 hours
Escribir dos preguntas a dos grupos sobre sus presentaciones y responder a las que se reciban	
Week 6	La población latina y la salud	
Presentar el status quo de la desigualdad en la salud y su resultado en la comunidad latina

Identificar los elementos dinamizadores y señalar estrategias e iniciativas.	Service 6-8 hours
Assignment 4 "Como hablar en silencio. Issues on language, culture, and identity of Central American in Los Angeles"
Final Project Outline Due
Journal 2 Due	
Week 7	Vivienda Digna. Gentrificación en California	Familiarizarse y crear conciencia sobre la falta de vivienda y la gentrificación en Los Ángeles y su impacto en la comunidad latina	Service 6-8 hours
Assignment 5 "Historias de gentrificación" Due	

Week 8	Latinos emprendedores.
¿Qué organización sin ánimo de lucro ustedes crearían?	Utilizar el universo léxico aprendido y las destrezas comunicativas escritas y orales para compartir perspectivas en español	Service 6-8 hours	
Week 9	No class. 30 min individual meetings with each group	Presentar el bosquejo de forma escrita y oral y aportar y recibir retroalimentación de tu profesora	Service 6-8 hours
Draft of Final Project Due	
Week 10	Final Oral Presentations	Desarrollar destrezas orales y auditivas a nivel avanzado en español a través la presentación y escucha de los proyectos finales
Utilizar el conocimiento cultural y lingüístico para contribuir a proyectos de justicia social	Service 6-8 hours
Journal 3 and Timesheet Due	


Course Policies:
Email response time: Please allow your instructor 24 hours to respond to any e-mail. The instructor reserves the right to not respond to messages during evening hours or during the weekend (Saturday and Sunday) or holidays.
Transportation: You are responsible for your own transportation to and from your service site. Lack of transportation will not be an acceptable excuse for not completing the required amount of hours of in-person service. If you are unable to do your volunteer work in person, a few of our community partners offer virtual volunteer opportunities.
COVID-19 Instructions: Ensuring a safer campus depends on each of us following the latest UCLA health and safety guidelines. While campus policies must be modified to address changing local, state, and national orders and guidance, the most current information is available at covid-19.ucla.edu.
At present, each of us must be fully vaccinated or have submitted an exception request and comply with weekly testing. Per the COVID-19 Response and Recovery Task Force, unvaccinated students with pending or approved exceptions must comply with twice-weekly testing.
I will do my best to support students. Contact me if you need to stay home, and we will make alternative arrangements for you to access class notes.
Be advised that refusal to comply with current campus directives related to COVID-19 mitigation may result in dismissal from the classroom and referral to the Office of Student Conduct. If you have any questions or concerns about UCLA’s COVID-19 protocol, go to https://covid-19.ucla.edu/information-for- students/; if you have any questions specific to this course, I am happy to talk further. Thank you for protecting your fellow Bruins!
Covid related absences: If you are experiencing symptoms, have a positive Covid test or have been told to isolate, please do not come to class. If you have symptoms, are positive or are isolating due to close contact, please inform your instructor. Your oral presentations may be rescheduled.
Guidance on protecting privacy and data during remote working and office hours through Zoom:

Note from the Office of Information Technology:
To the Campus Community:
The University protects the privacy of faculty, students, and staff while working or participating in academic programs. Use of remote delivery software and technologies heightens the criticality of privacy and the need to use the least invasive means of engaging in these alternative methods of conducting our activities. Existing law and policy that address privacy remain in effect.
All faculty, staff and students must follow these principles:
Video or audio recording of a lecture is permitted but only with advance notice and opportunity to opt out of video/audio participation. To enable the recording feature, the host must first download and install the native Zoom app on their computer.
Where recording is permitted, it is permitted only by the host (typically instructor or meeting chair). Students in a class and/or meeting participants and any student-hosted meetings are prohibited from recording of any kind. Disability accommodations relating to recordings are addressed in the specific guidance in the link referenced below.
Video or audio recording (including taping, recording, photographing, screen capture and other methods of capture) for purposes other than instruction is prohibited absent a strong rationale and only if the host provides advance notice and opportunity to opt out of video/audio participation.
During video conferencing, there is a chat function that permits participants to ask questions and engage in dialogue as the class or meeting proceeds. Recording, including photographing, screen capture, or other copying methods of chat exchanges is prohibited except by the instructor or meeting chair when advance notice is provided.
Online advising can occur via chat, audio, or video conferencing using Zoom or other approved software tools, or by phone. Sessions should not be recorded; rather, the advisor should log notes in the customary fashion.
Zoom is the primary approved software tool for remote live and recorded academic sessions and meetings. For the specific guidance on how to appropriately use Zoom while affording privacy protection, including approved notification language that provides opt out options, see Guidance on Protecting Privacy and Data During Remote Working and Teaching Using Zoom while COVID-19 Modifications are in Effect.
Late work: All assignments must be turned in by the specified due dates – no late work will be accepted (except in cases where students can present written documentation of illness or other serious, exceptional circumstances).
Academic Integrity: UCLA is a community of scholars. In this community, all members including faculty, staff and students alike are responsible for maintaining standards of academic honesty. As a student and member of the University community, you are here to get an education and are, therefore, expected to demonstrate integrity in your academic endeavors. You are evaluated on your own merits. Cheating, plagiarism, collaborative work, multiple submissions without the permission of the professor, or other kinds of academic dishonesty are considered unacceptable behavior and will result in formal disciplinary proceedings usually resulting in suspension or dismissal.
Additional information can be found on the website for the Office of the Dean of Students: https://deanofstudents.ucla.edu/student-conduct-code

Remember: There are many alternatives to academic dishonesty!
Seek out help – meet with your TA or Professor, ask if there is special tutoring available.

Drop the course – can you take it next quarter when you might feel more prepared and less pressured?
Ask for an extension – if you explain your situation to your TA or Professor, they might grant you an extended deadline.
See a counselor at Student Psychological Services, and/or your school, college or department – UCLA has many resources for students who are feeling the stresses of academic and personal pressures.

Resources:
Accessibility:
Center for Accessible Education (CAE)
The University provides services to students with disabilities and should any student require disability- related accommodations, they should contact the Center for Accessible Education at: www.cae.ucla.edu
TDD (310) 825-1501 or in person at Murphy Hall A255
If you are already registered with the Center for Accessible Education (CAE), please request your Letter of Accommodation in the Student Portal. If you are seeking registration with the CAE, please submit your request for accommodations via the CAE website. Students with disabilities requiring academic accommodations should submit their request for accommodations as soon as possible, as it may take up to two weeks to review the request. When possible, students should contact the CAE within the first two weeks of the term as reasonable notice is needed to coordinate accommodations. Please note that your instructor will not discuss or offer any accommodations that have not been documented and recommended officially by the University.
Similar to disability-related accommodations for courses, students with disabilities can request accommodations for Community Engagement sites connected to seminars through the UCLA Center for Community Engagement. Reasonable accommodations for these types of experiences are determined through engagement of the student, the CAE, the faculty, and the site supervisor, on an individualized basis. If you are not yet registered with the CAE and would like to request accommodations for these settings, please proceed to submit a request for accommodations on the CAE website. If you are already registered with the CAE and would like to request accommodations for these types of settings, please email your Disability Specialist directly to discuss your requests.
To ensure timely processing of requests, students should submit a request for disability-related accommodations with supporting medical documentation as soon as courses are finalized or as soon as possible. Requests submitted after the quarter begins will still be considered, but accommodation options may be limited.
Providing feedback to me: I encourage your feedback at any time throughout the quarter about things that are helping you learn, or things that aren’t helping. Please communicate with me or with your TA if there are ways that we can improve the course to better support student learning.
Personal Problems: I understand that sometimes life makes it difficult to focus on schoolwork. If you are having a personal problem that affects your participation in this course, please talk to me to create a plan. Please do not wait until the end of the quarter to share any challenges that have negatively impacted your engagement and academic performance. The sooner we meet, the more options we will have available to discuss to support your overall academic success. If you are not comfortable speaking with me directly,

please utilize the other student resources provided below in order to understand how to best approach success in this course given your personal needs as soon as possible.
UCLA provides resources if you are feeling overwhelmed and need personal and/or academic assistance. Please see the Red Folder REV2020 web for more information.
Navigating Student Services
UCLA’s new Student Affairs Guidebook gathers key resources for graduate and undergraduate students related to living and learning during these tumultuous times: https://www.studentaffairs.ucla.edu/guidebook. Students can always consult the Student Care Managers program website for information about supportive services, including information about confidential one- on-one consultation:
http://www.studentincrisis.ucla.edu/Who-can-Help.

Basic Needs/Food Resources
If you find yourself having difficulty accessing meals and/or groceries, go to this link for information on a variety of on- and off- campus resources available to students: https://basicneeds.ucla.edu/.

LGBTQIA Resources
The UCLA LGBT Campus Resource Center (www.lgbt.ucla.edu) provides a range of services supporting intersectional identity development as well as fostering unity, wellness, and an open, safe, and inclusive environment. UCLA also has a range of policies and services especially relevant to transgender students here:	www.lgbt.ucla.edu/Trans-At-UCLA.

Mental Health
As a student you may experience a range of issues that can cause barriers to learning, such as strained relationships, increased anxiety, alcohol/drug problems, depression, difficulty concentrating and/or lack of motivation. These mental health concerns or stressful events may lead to diminished academic performance or reduce a student's ability to participate in daily activities. UC offers services to assist you with addressing these and other concerns you may be experiencing. If you or someone you know are suffering from any of the aforementioned conditions, consider utilizing the confidential mental health services available on campus. I encourage you to reach out to the Counseling Center for support (www.counseling.ucla.edu and 310-825-0768, available 24/7). An on campus counselor or after-hours clinician is available 24/7.
TITLE IX
Title IX prohibits gender discrimination, including sexual harassment, domestic and dating violence, sexual assault, and stalking. If you have experienced sexual harassment or sexual violence, you can receive confidential support and advocacy at the CARE Advocacy Office for Sexual and Gender-Based Violence, 1st Floor Wooden Center West, CAREadvocate@careprogram.ucla.edu, (310) 206-2465.
In addition, Counseling and Psychological Services (CAPS) provides confidential counseling to all students and can be reached 24/7 at (310) 825-0768.
You can also report sexual violence or sexual harassment directly to the University's Title IX Coordinator, 2241 Murphy Hall, titleix@conet.ucla.edu, (310) 206-3417. Reports to law enforcement can be made to UCPD at (310) 825-1491.

Faculty and TAs are required under the UC Policy on Sexual Violence and Sexual Harassment to inform the Title IX Coordinator should they become aware that you or any other student has experienced sexual violence or sexual harassment.
“This syllabus is intended to give you guidance in what may be covered during the quarter and will be followed as closely as possible. However, the professor reserves the right to modify, supplement and make changes as the course needs arise.”


Thank you for your participation in this course!
