# Supplementary material for: Increasing Accessibility to Neuroscience through Translation: Going beyond the English Language
Source: eNeuro. 2024 Jan 10;11(1):ENEURO.0392-23.2023. doi: 10.1523/ENEURO.0392-23.2023 (PMC11078105; doi:10.1523/ENEURO.0392-23.2023)
Supplement: Extended Data 2-3 — Alphabetical list of articles translated between April of 2021 and June of 2023. A total of 102 articles have been translated in a span of 7 academic quarters (spring 2021 – spring 2023; 26 months). A large range of topics (article titles), spanning multiple unique subject areas (“topic tags”, used to index content in the Knowing Neurons website) have been translated. All articles in their original English version can be found at https://knowingneurons.com, and all of the translated versions in Spanish can be found at https://knowingneurons.com/es. Download Extended Data 2-3, DOCX file. [file eneuro-11-ENEURO.0392-23.2023-s003.docx]

| ***English Article (Title)*** | ***Quarter and year*** | ***Topic tags*** |
| --- | --- | --- |
| A Tale of Two Autisms | Spring 2023 | neurological & psychiatric disorders, sensation & perception |
| Addiction Basics | Spring 2021 | mental health, neurological & psychiatric disorders |
| Aging | Spring 2021 | aging, neurological & psychiatric disorders |
| Attention | Spring 2021 | consciousness |
| Biomarkers for Alzheimer’s Disease | Winter 2023 | cognition, neuroscience technologies |
| Blondes or Brunettes: It’s All About Conditioning | Winter 2023 | did you know?, learning & memory |
| Brain, Healthy Fats, and the Importance of Fitting Into Your Genes | Spring 2021 | big ideas, brain development, culture |
| Can We Trust MRI Research? It's Complicated. | Fall 2021 | neuro news, neurological & psychiatric disorders |
| Cerebral Organoids | Spring 2021 | brain basics, neuro primer |
| Chromosome Silencing: Turning Off Genes in Down Syndrome | Fall 2022 | neuro news, neurological & psychiatric disorders |
| CLARITY | Fall 2022 | neuro news |
| Depression: the Role of Chronic Inflammation | Winter 2022 | brain development, mental health, neurological & psychiatric disorders |
| Dialogues in Music Therapy and Social Neuroscience: An Interdisciplinary Approach to Clinical Progress | Spring 2023 | big ideas, brain development, neurological & psychiatric disorders |
| Do People with Dyslexia Read and Write Backwards? | Winter 2022 | brain development, language |
| Does Free Will Exist? | Winter 2022 | big ideas, interviews, philosophy |
| Empathy: Building Social Interactions by Linking Our Emotional Lives | Spring 2023 | big ideas, sensation & perception |
| Evidence-Based Solutions to the Mass Incarceration Crisis: Neuroscience and Cash Bail | Spring 2021 | big ideas, science policy |
| Excitation and Inhibition: the Yin and Yang of the Brain | Spring 2022 | neurological & psychiatric disorders |
| Face Perception in Autism Spectrum Disorder | Spring 2023 | brain development, neurological & psychiatric disorders |
| Final Decision? Why the Brain Keeps on Changing its Mind | Winter 2022 | brain basics |
| Focused Ultrasound: A Stimulating New Strategy to Treat Severe Brain Damage | Spring 2021 | big ideas, consciousness, neurological & psychiatric disorders, sensation & perception |
| Forever Young: The Story of Patient H. M. | Winter 2023 | did you know?, learning & memory |
| Garbage Smells Green and Gunshots are Rainbows | Spring 2022 | consciousness, sensation & perception, videos |
| Glia | Fall 2021 | aging, brain basics, brain development, neuro primer, neurological and psychiatric disorders |
| Gut Feelings: the Connection With the Brain | Winter 2022 | big ideas |
| Hacking your Brain with Smart Drugs | Fall 2022 | sensation & perception |
| Heightened Senses: Cross-Modal Neuroplasticity | Winter 2023 | learning & memory, neuro news, popular articles, sensation & perception |
| Hormones and Body Weight | Spring 2021 | brain basics, neuro primer |
| How Brain Stimulation Can Boost Memory If Paired with Learning | Spring 2021 | learning & memory |
| How Do we Know? The Value of Scientific Models. | Spring 2022 | big ideas, philosophy, popular articles |
| How Floating in Darkness Takes the Body off the Mind | Spring 2022 | big ideas, consciousness, evolution of cognition, neuro news, philosophy |
| How Neuroimaging Changes Our View of Science & Humanity | Spring 2022 | big ideas, neuroscience art, sensation & perception |
| How to Give Thanks Like a Neuroscientist | Spring 2022 | brain basics |
| How Weight Lifting Gets the Brain in Shape | Spring 2023 | cognition, learning & memory, movement |
| Imaging the Brain with Sculpted Light | Spring 2023 | neuro news, neuroscience technologies |
| Invisible Modulators of Fear: How the Gut Microbiome Alters Fear Extinction | Spring 2021 | big ideas, sensation & perception |
| Is Neuroimaging Just Modern Phrenology? | Spring 2021 | big ideas, German (multilingual), philosophy |
| Is Self-Awareness Humanity´s Greatest Trait? | Winter 2023 | did you know?, learning & memory |
| Keeping Memories Fresh by Keeping Glutamate in Check | Winter 2023 | aging, learning & memory, neuro news |
| Language: Your Connection to Others | Spring 2021 | language, sensation & perception |
| Learning about Language from Birdsong | Fall 2021 | brain basics, learning & memory |
| Lighting up the Brain with Optogenetics | Spring 2022 | brain basics, neuro news, neurological & psychiatric disorders, video |
| Mapping Brain Connectivity Using Graph Theory | Fall 2022 | did you know?, neuroscience technologies |
| Neuro Primer: Working Memory Capacity | Winter 2023 | learning & memory, neuro primer |
| Neurodevelopment | Spring 2021 | brain basics, neuro primer |
| Neuronal Communication: Electricity and Neurotransmitters | Fall 2021 | brain basics, sensation & perception |
| Neuronal Migration: A "Sliding Door" for the Future Brain | Spring 2021 | brain development |
| Neuroscience Education in the Time of COVID-19: An Interview with Dr. Megan Peters about Neuromatch Academy | Spring 2021 | artificial intelligence, big ideas, collaborations, interviews, neuro news |
| No Pain, No Gain: Commensal Bacteria Help Chemotherapy Through Painful Side Effects | Spring 2022 | neuro news |
| No, You're Not Left-Brained or Right-Brained | Spring 2022 | brain basics, did you know? |
| One Small Map for Fruit Flies, One Giant Leap for Neuroscience | Fall 2022 | neuro news |
| Optogenetics: An Illuminating Tool with A Bright Future | Spring 2021 | big ideas, learning & memory, movement |
| Organs-on-chips: Growing Miniature Human Organs | Spring 2021 | neuroscience technologies |
| PET Imaging: The Real Positronic Brain? | Spring 2022 | neuroscience technologies |
| Reading: The Brain's Best Hijacker | Fall 2021 | brain development, learning & memory, sensation & perception |
| Reconciling the Past with Pills: A New Approach to PTSD Treatment | Winter 2023 | learning & memory, neuro news, neurological & psychiatric disorders |
| Remote Control of the Brain Is Coming: How Will We Use It? | Spring 2021 | big ideas |
| Retinal Prostheses: Restoring Vision to the Blind | Fall 2022 | neuro news, sensation & perception |
| Reverse Engineering the Brain | Spring 2021 | artificial intelligence, big ideas, consciousness, German (multilingual) |
| Schizophrenia in a Vial? The Story of Ketamine | Winter 2022 | brain development, neurological & psychiatric disorders, sensation & perception |
| Seeing Invisible Colors: Part II | Spring 2022 | sensation & perception |
| Self Reflected: the Best of Neuroscience and Art | Fall 2022 | Interviews, neuroscience art, sensation & perception |
| Sex Differences in the Neural Control of Thermogenesis: An Interview with Dr. Stephanie Correa | Spring 2021 | collaborations, interviews, neuro news |
| Sleep | Spring 2021 | consciousness, sleep, neurological & psychiatric disorders, sensation & perception |
| Sleep Paralysis: What Is It and What Causes It? | Spring 2021 | consciousness, sleep, neurological & psychiatric disorders, sensation & perception |
| Stimulating Neural Circuits with Magnetism | Spring 2023 | did you know?, neuroscience technologies |
| Surfing Brainwaves with EEG: A Classic Tool for Recording Temporal Brain Dynamics | Spring 2022 | brain basics, did you know?, neuroscience technologies |
| The AMI Procedure: Gateway to a Cyborg Future | Fall 2022 | artificial intelligence, big ideas, evolution of cognition, neuro sci-fi |
| The Blood Brain Barrier- Is it Really All it's Described to Be? | Spring 2021 | big ideas |
| The Brain's Building Blocks: of Protons and Voxels | Fall 2021 | brain basics, neuroscience technologies |
| The Default Mode Network and Depression | Spring 2023 | mental health, neurological & psychiatric disorders |
| The Departure of Skill Memories from Motor Cortex: Deeper Directions for Neuroscience | Spring 2023 | learning & memory, movement, neuro news |
| The emotional mechanics of the robot human interaction | Spring 2022 | neuro news |
| The Epigenetic Legacy of Trauma | Spring 2022 | neurological & psychiatric disorders |
| The Fatal Relationship Between Firearm Policy and Brain Development | Spring 2023 | brain development, mental health, science policy |
| The Fugue of Life: Why Complexity Matters in Physiology and Neuroscience | Fall 2021 | big ideas, neurological & psychiatric disorders, popular articles |
| The inescapable nightmare of fatal familial insomnia | Spring 2022 | neurological & psychiatric disorders, sleep |
| The Law of Attraction, the Placebo Effect, and the Immune-Brain Connection | Winter 2023 | big ideas, did you know?, neurological & psychiatric disorders |
| The Life and Times of the 10% Neuromyth | Spring 2023 | brain basics, neuro sci-fi |
| The Microbes that Make Us Human | Spring 2022 | neurological & psychiatric disorders |
| The Neuroscience of Star Wars | Spring 2023 | did you know?, neuro sci-fi, neuroscience technologies |
| The Nocebo Effect | Spring 2021 | big ideas, mental health, neurological & psychiatric disorders, neuro news |
| The Past and Promise of Deep Brain Stimulation | Spring 2022 | neurological & psychiatric disorders |
| The Promise of Service Dogs in Autism | Spring 2023 | big ideas, brain development, neurological & psychiatric disorders, sensation and perception |
| The Smell of the Good Ol’ Days | Winter 2023 | learning & memory, neuro news |
| The Tenets of Tauists | Spring 2023 | learning & memory, neuro news, neurological & psychiatric disorders |
| The Turing test: Is That Human or Machine? | Spring 2022 | artificial intelligence |
| The Ultimate Thought Experiment Part III: Flowers for Algernon | Spring 2022 | big ideas, neuro sci-fi, neurological & psychiatric disorders, philosophy |
| The Wisdom of the Sloth: Is Sleep a Lost Virtue? | Spring 2022 | culture, sleep |
| This Is Your Brain on Twitter: The Neuroscience of Social Media | Winter 2023 | bias, big ideas, brain basics |
| Tick, Tock: Your Brain's Inner Clock | Winter 2022 | sensation & perception |
| Turning On A “Photoswitch” Helps Blind Mice See the Light | Spring 2023 | neuro news, neuroscience technologies, sensation & perception |
| Unwrap the Gift from the Deadliest Toxin | Fall 2022 | movement |
| Vestibular System | Spring 2021 | movement, sensation & perception, space science |
| Vocabulary Retention In Adult Language Learners | Spring 2022 | big ideas, brain development, evolution of cognition, language |
| What Happens If You Stick Your Head in a Particle Accelerator? | Fall 2022 | big ideas |
| What I Learned from Tracking My Sleep with Fitbit for Two Months | Spring 2021 | collaborations, sleep |
| What is the cerebellum? | Fall 2021 | brain basics, movement, sensation & perception |
| Why Does Music Make Us Feel So Much? | Winter 2022 | big ideas |
| Why Mosquitoes Love Us | Winter 2023 | did you know?, sensation & perception |
| You're Not as Rational as You Think: Political Philosophy and the Science of Irrationality | Fall 2022 | big ideas |
| Your gut is closer to your brain than you think! | Fall 2022 | neurological & psychiatric disorders |

**Alphabetical list of articles translated between April of 2021 and June of 2023**. A total of 102 articles have been translated in a span of 7 academic quarters (spring 2021 – spring 2023; 26 months). A large range of topics (article titles), spanning multiple unique subject areas (“topic tags”, used to index content in the Knowing Neurons website) have been translated. All articles in their original English version can be found at <https://knowingneurons.com>, and all of the translated versions in Spanish can be found at <https://knowingneurons.com/es>.
