# Supplementary material for: Increasing Accessibility to Neuroscience through Translation: Going beyond the English Language
Source: eNeuro. 2024 Jan 10;11(1):ENEURO.0392-23.2023. doi: 10.1523/ENEURO.0392-23.2023 (PMC11078105; doi:10.1523/ENEURO.0392-23.2023)
Supplement: Extended Data 2-4 — Lesson Plan Development Template. Students are provided with this template to develop their lesson plans with the supervision of their professors in the Department of Spanish and Portuguese and Brain Research Institute. Download Extended Data 2-4, DOCX file. [file eneuro-11-ENEURO.0392-23.2023-s004.docx]

**Lesson Plan Development Template.**


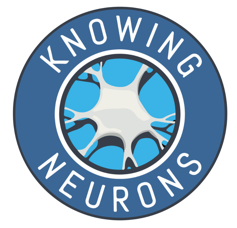

**Title:** *Add the title of your lesson plan*

**KN Article Title:** *Please specify which Knowing Neurons article this lesson plan is linked to*

**Difficulty level:** *Please specify the targeted difficulty level of this lesson (e.g basic-intermediate-advanced)*

*Please try to keep this lesson plan between 50 mins - 1 hour, so it can be implemented in regular school classrooms. Keep in mind that within this time frame teachers should be able to introduce the topic, students should conduct an activity and, lastly, there should be some time at the end of the lesson for closing remarks.*

*Ideally, the activity conducted by the students would represent a substantial part of the lesson time (ideally 25-30 minutes in a 1 hour long lesson). The presentation and lecture portion should ideally be less than half of the allotted time.*

*Through this collaboration, at Knowing Neurons we are interested in evaluating the impact of these lesson plans on the students. Therefore, we want to administer a questionnaire to the students in order to understand how their participation in this lesson shapes their views on science and themselves as scientists. Furthermore, we want to evaluate the impact that we are making so we can improve these lesson plans in the future and, for that, we need feedback from the students!*

*In order to gather this information we have created standardized questionnaires to be administered to the students. Please ensure that you have discussed giving this questionnaire to the students with the teacher ahead of time so that it can be administered 1 week prior to your arrival at the school.*

- Pre-questionnaire (must be completed 1 week before the lesson)
- Post-questionnaire (must be competed 1 week after the lesson)

1. **Overall Lesson goals:**

*Please provide 3 general goals of this lesson plan.*

- Example: Students will learn about memory, students will be able to construct an experiment, etc


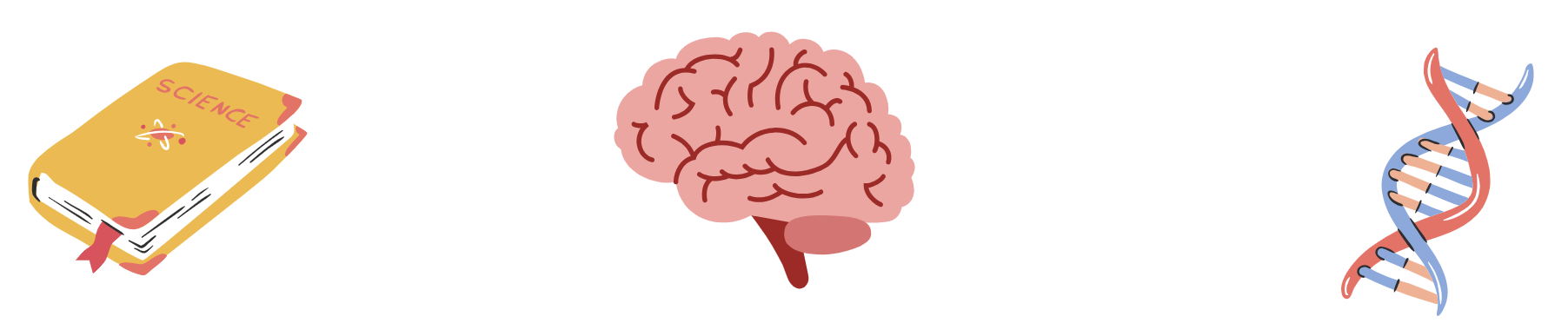


1. **Overall Lesson Learning objectives**

Please provide*a list of 5-10 specific, measurable goals that an educator can implement or test in the classroom. You can also add whether those goals will be learned during the presentation section or whether they should be gained during the activity part.*

- *For example: students should be able to highlight one region of the brain involved in memory processes, students will be able to identify one role of bacteria in the body, students will be able to construct an experiment to assess memory).*

1. **Materials (visual and physical)**

*Please add a brief list of the material teachers will need to conduct this lesson plan (including the PowerPoint presentation)*

1. **Presentation**

*This section  introduces the lesson topic for the specific article.*

*Please, remember that an educator from anywhere in the world may want to use this lesson plan in their classrooms, therefore you should provide detailed, step by step instructions and background on each of the slides presented.*

- Please add a bulleted list of speaker notes (with citations to sources and papers where necessary) on each slide of your presentation.

*An educator should be able to read the lesson plan and know exactly what to do and say. The presentation should include:*

- *At least 1 slide introducing Knowing Neurons*
  - *Feel free to use graphics and pictures from the Knowing Neurons Website, and reach out to the Knowing Neurons team if you’d like help on how to approach this slide.*

*The following points are suggestions that could be covered in the presentation and used as a guideline:*

- Introduction to the scientific concepts covered in the article
- Introduction to the article itself
- Conclusion linking the scientific content learned and the article itself

1. **Presentation key points**

*Please specify an outline of the key concepts covered in the presentation of your lesson plan in detail. e.g:*

- Brief introduction to the field of neuroscience
- Introduction to the role of bacteria in the gut and brain
- Introduction to the research presented in the associated Knowing Neurons article


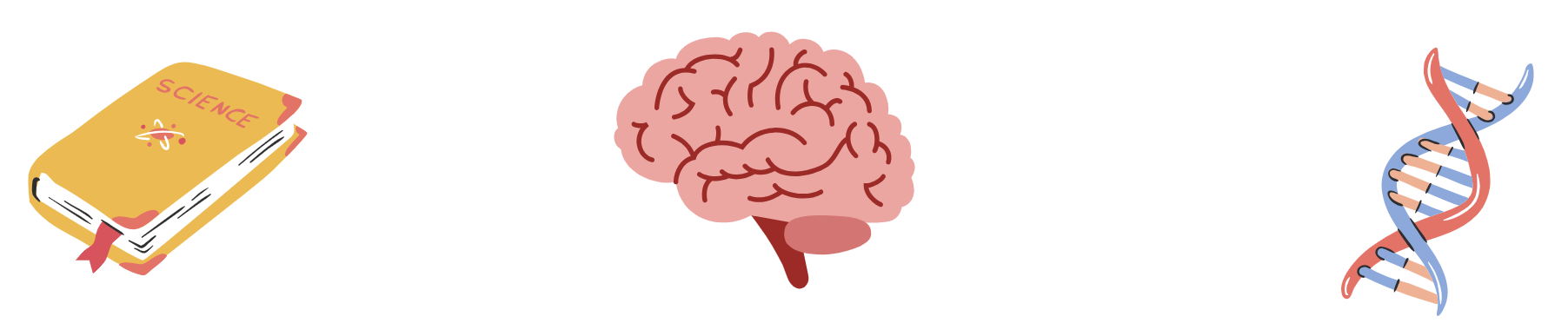


1. **Activity**

*Please include detailed instructions on what the activity will be and how will this activity take place  as well as a detailed description and breakdown of the timings of the activity.*

- *Example: 2 Minutes: Form groups, 5 minutes: introduce the activity, 15 minutes: conducting activity, 5 minutes: clean up...*

1. **Materials needed for the activity**

*Please provide a detailed list of materials needed for the classroom. If any materials need to be printed or accessed from outside (e.g., Internet) please also provide a digital copy of the material itself.*
